# Dietary zinc intake and mortality in patients with intestinal-type gastric cancer: A prospective cohort study in Korea

**DOI:** 10.3389/fonc.2022.947405

**Published:** 2022-11-07

**Authors:** Jung Hyun Kwak, Chan Hyuk Park, Chang Soo Eun, Dong Soo Han, Yong Sung Kim, Kyu Sang Song, Bo Youl Choi, Hyun Ja Kim

**Affiliations:** ^1^ Department of Food and Nutrition, Gangneung-Wonju National University, Gangneung-si, South Korea; ^2^ Division of Gastroenterology, Department of Internal Medicine, Hanyang University Guri Hospital, Guri-si, South Korea; ^3^ Functional Genomics Institute, PDXen Biosystems Co., ETRI Convergence Commercialization Center, Daejeon, South Korea; ^4^ Department of Pathology, Chungnam National University College of Medicine, Daejeon, South Korea; ^5^ Department of Preventive Medicine, Hanyang University College of Medicine, Seoul, South Korea

**Keywords:** zinc, intestinal-type, cohort study, gastric cancer, mortality

## Abstract

**Purpose:**

Current evidence regarding the association between zinc intake and gastric cancer (GC)-specific survival in patients with intestinal-type GC is lacking. Therefore, this cohort study investigated the association between zinc intake and GC mortality through follow-up on GC death among patients with intestinal-type GC and whether these effects differ according to the source of zinc intake.

**Methods:**

A total of 185 patients with intestinal-type GC were enrolled from two hospitals between 2002 and 2006. Their survival or death was prospectively followed up until December 31, 2016, through a review of medical records and telephone surveys.

**Results:**

A total of 178 patients were included and analyzed. The median follow-up period was 7.3 years. In the fully adjusted models, the highest tertile of total zinc intake showed a significantly lower GC mortality than the lowest tertile (hazard ratio, 0.22; 95% confidence interval: 0.08–0.64). In addition, the tertile of total zinc intake showed a dose-response association with GC mortality (p=0.015). Analysis of the source of zinc intake revealed that when zinc intake from staples (rice and noodles), animal, and plant food sources were combined, the results were similar to those of total zinc intake and GC mortality.

**Conclusion:**

Zinc intake through various foods may be effective in reducing GC mortality by achieving balance with other nutrients. Our results suggest that zinc improves the survival of patients with intestinal-type GC in Korea.

## Introduction

According to Statistics Korea, the estimated crude mortality rate of gastric cancer (GC) was 14.9 per 100,000 persons in 2019 (1), which makes it the most common cancer in Korea. According to the Lauren classification, the main histological types of GC are the intestinal and diffuse types (2). Intestinal-type GC occurs more frequently than diffuse-type GC; furthermore, the incidence rate of intestinal-type GC is higher in older male patients and is more closely related to environmental factors, such as diet (3). According to previous studies, positive or negative associations with diet are indeed prominent in intestinal-type GC (4, 5). Therefore, it is essential to determine the dietary factors that affect mortality in patients with intestinal-type GC.

Previous studies have reported that dietary factors, such as vitamins, minerals, and natural compounds help reduce the risk of chronic diseases because the major mechanisms of chronic diseases, such as obesity, hypertension, and cardiovascular disease are related to oxidative stress and inflammatory responses (6, 7). However, not all the reports were consistent. According to previous studies, foods containing zinc, such as nuts (8) and *Nigella sativa* plants (9), are effective against chronic diseases, such as diabetes and cardiovascular disease; however, the consumption of eggs (10) had no effect on hypertension. Huang et al. reported that their study’s results were not sufficient to prove the effects of vitamin and mineral supplementation on cancer and chronic diseases (11).

Zinc is an essential trace element, the second most abundant element in the body, and the most abundant intercellular trace mineral (12). Zinc acts as a component of 300 Zn metalloenzymes, which are required for the metabolism of macronutrients and the clearance of reactive oxygen species. In addition, zinc regulates gene transcription (12) and maintains immune functions (13). Zinc has antioxidant properties and is a known protective agent against cancer (14). Several epidemiological and review studies (15–19) have investigated the association between zinc intake and GC risk. However, the results were limited to the incidence of GC and were inconsistent. In the literature, some studies (15–17) suggest that zinc intake is inversely associated with GC risk, whereas other studies (18, 19) found no association with GC risk.

Zinc is closely related to immune function, acts as a tumor suppressor in GC (20), and is thought to have beneficial effects on GC incidence and mortality. In a systematic review and meta-analysis, Li et al. reported that zinc intake was significantly associated with GC incidence in Asia, but not in America and Europe (17). They discussed that the reason for the difference in geographic region might be that food sources of zinc vary among ethnic groups. In the Western diet, the main food source of zinc intake is from animal sources, such as red meat, poultry, and dairy foods, whereas in Asia, most zinc intake is obtained from grains and tends to consume more fish and less than red meat (21, 22). However, studies on the effects of zinc intake from food sources on GC mortality are rare. Additionally, current evidence regarding the association between zinc intake and GC-specific death in patients with intestinal-type GC is lacking.

The novelty of our study is that zinc intake was divided into animal food groups, plant food groups, and staple foods according to food sources, and these groups were analyzed individually or in various combinations. In addition, we selected and analyzed only the intestinal type of GC, which is highly prevalent in Korea and known to be highly related to diet.

Therefore, this cohort study investigated the association between zinc intake and GC mortality by assessing GC death among patients with intestinal-type GC, and examining whether these associations differ according to the source of zinc intake.

## Method

### Study design and patients

We created a prospective cohort of 508 patients aged ≥20 years who were newly diagnosed with GC at the Chungnam National University Hospital and Hanyang University Guri Hospital between March 2002 and September 2006. Patients who voluntarily wanted to participate after the doctor explained the study to them were recruited in the first stage (March 2002 to August 2003) and the second stage (October 2003 and September 2006). Patients were followed-up from diagnosis until the date of death from GC or at the end of follow-up (December 31, 2016). The patients were diagnosed histologically based on the World Health Organization classification of tumors of the digestive system (23) as follows: gastroscopy was performed by a gastroenterologist, and the final diagnosis of GC was confirmed by a pathologist through a biopsy. The histological subtypes of GC were classified according to the Lauren’s classification (2). The purpose of this study was to confirm the association between zinc intake and GC mortality in patients with intestinal-type GC. We selected only 185 patients with intestinal-type GC. Intestinal type GC was defined based on the adherence of tumor cells, as well as the formation of glands and tubular structures. Among the 185 patients, 7 with abnormal energy intake (n=4, <500 kcal or n=3, >5000 kcal) were excluded. All patients provided written informed consent to voluntarily participate in the study, which was approved by the institutional review board (IRB) of Hanyang University Guri Hospital (IRB no. 2003-4). For follow-up, additional approval was obtained from the IRB of Chungnam National University Hospital (IRB no. CNUH 2017-12-039), and the IRB of Hanyang University Guri Hospital (IRB no. 2018-01-021-001).

### Data collection

The questionnaire included questions on sociodemographic characteristics, such as sex, age, and education level; anthropometric factors, such as height and weight; behavioral factors, such as smoking status and alcohol consumption; clinicopathological factors; and dietary factors. Regarding clinicopathological factors, including cancer location and size, gross type, histological type, pathological tumor-node-metastasis (TNM) stage, surgery, and chemotherapy, the gastroenterologist checked the patients’ medical records during the study period. Pathological data of all patients were re-reviewed in March 2018 to apply the cancer staging system according to the 8^th^ edition of the American Joint Committee on Cancer (AJCC) TNM classification for GC. *Helicobacter pylori* (*H. pylori*) infection was measured using a Campylobacter-like organism test kit with high sensitivity and specificity (24) (Product no: 60480; Kimberly-Clark/Ballard Medical Products, Draper, UT, USA). A family history of GC included first-degree relatives. We grouped the participants into three categories based on their body mass index (BMI) according to the Asia-Pacific classification (25):1) underweight and normal weight, ≤22.9 kg/m^2^; 2) overweight, 23.0–24.9 kg/m^2^; and 3) obese, ≥25 kg/m^2^.

### Dietary data

Nutrient and food intakes were assessed using a quantitative food frequency questionnaire (FFQ). All questionnaires were investigated by well-trained interviewers, and the patients were asked to recall their usual dietary intake from 3 years earlier in order to assess past dietary intake. The duration of dietary recall was set to 3 years because patients may have changed their dietary habits owing to signs of poor health status before they were diagnosed with GC. In nutrition-related cancer studies, remote dietary intake may be more important than recent dietary intake owing to the long latency of cancer. For the first stage, the FFQ included 102 food or dish items and investigated the intake period during 1-year, average frequency of food consumption per month, week, or day, and one serving size. In the second stage, the FFQ included 115 food or dish items, and the frequency of food consumption was investigated in nine categories (1): never or less than once a month; (2) 1–3 times a month; (3) 1 time a week; (4) 2–4 times a week; (5) 5–6 times a week; (6) 1 time a day; (7) 2–3 times a day; (8) 4–5 times a day; and (9) ≥6 times a day and one serving size. The intake of one serving size of each food item was investigated in an open-ended manner using a general unit suitable for each food or dish (e.g., a bowl, plate, or piece). Most survey items at each stage were similar. However, items with some differences were analyzed after unifying the survey items in the first stage as the standard of the second stage. The daily intake of each food or dish was calculated by multiplying the daily frequency by the serving size. The daily intake of total energy and nutrients from food or dish items was estimated using the Korean Foods and Nutrients Database (26). Heme iron was calculated by applying a factor of 0.4 to animal foods, including red meat, poultry, fish, and animal organs (27, 28). Energy-adjusted nutrient intake was adjusted for the total energy intake using the residual method (29). Since there was a slight difference between the FFQs for the first and second stages, the tertiles of zinc intake were applied according to each stage. The lowest tertile of zinc intake was used as a reference. The median values of each tertile category of Zn intake were used as continuous variables to test for trends. We used a slightly modified version of the validated FFQ used in our earlier study (30). To analyze the source of zinc intake, zinc intake from staple foods, including rice and noodles; animal food groups, including beef, pork, chicken, fish, and dairy products; and plant food groups, including beans, fruits, kimchi, and vegetables were analyzed for individual food groups or combinations.

### Follow-up

Patients were followed-up from diagnosis until the date of death from GC or at the end of follow-up. A total of seven follow-up investigations (2003, 2004, 2005, 2008, 2011, 2012, and 2017) were conducted to confirm death due to GC, and the end date of the last follow-up was December 31, 2016. The overall follow-up rate was 73.0%, and the date and cause of death were confirmed by the examination of medical records. However, if this could not be confirmed in medical records, we conducted telephone surveys. In instances in which the exact date of death was unclear from the telephone survey, the median value of the month of death was substituted. The final status of the cases was classified into five categories (GC death, non-GC death, GC recurrence, survival, and follow-up failure). The survival period was calculated from the date of surgery (patients who had not undergone surgery were based on the date of diagnosis) to the last follow-up or censoring date of each patient.

### Statistical analyses

General patient characteristics are presented as numbers with proportions for categorical variables, and as means and standard deviations for continuous variables. Categorical variables were compared using the chi-squared test. The mean differences between the zinc intake groups were compared using analysis of variance. Survival analysis was performed using the Kaplan–Meier method with a log-rank test. Cox proportional hazard regression analysis was performed to assess risk factors for GC mortality. We analyzed factors that influence the prognosis of GC mortality and selected age, sex, alcohol consumption, GC stage, and adjuvant chemotherapy as covariates that showed significant results. In addition, additional adjusted covariates (i.e., smoking, level of education, BMI, registered hospital, family history of GC, *H. pylori* infection, and surgery) that are known to affect the prognosis of GC mortality were selected through a literature review. Level of education was adjusted instead of socioeconomic status because level of education and economic status were highly correlated (31, 32). Model I was adjusted for age, sex, adjuvant chemotherapy (no or yes), and GC stage (I, II, III, IV, or unknown). Model II was further adjusted for BMI (≤22.99 kg/m^2^, 23.0–24.99 kg/m^2^, ≥25 kg/m^2^, or missing), level of education (≤ elementary school or none, middle or high school, ≥ college or higher, or unknown), smoking status (never, past, or current smokers), alcohol drinking (never, past, <20 g/day for women or <40 g/day for men, or ≥20 g/day for women or ≥40 g/day for men), hospital (Chungnam University Hospital or Hanyang University Guri Hospital), first-degree family history of GC (no or yes), *H. pylori* infection (negative, positive, and not performed), and surgery (no or yes). Model III was further adjusted for energy-adjusted heme iron intake (continuous). Events were defined as GC death, and other cases of non-GC death, GC recurrence, survival, and follow-up failure were coded as censored cases. The risk of death was presented as hazard ratio (HR) with a 95% confidence interval (95% CI). The proportional hazards assumption was tested by creating an interaction term between zinc intake (median of each tertile) and the follow-up time (continuous). The interaction term was not statistically significant; thus, we concluded that the proportional hazard assumption was met. Statistical significance was set at P <0.05. All statistical analyses were performed using SAS version 9.4 (SAS Institute Inc., Cary, North Carolina, USA).

## Results

### General characteristics

In the 178 patients with intestinal-type GC in this study, the total person-years were 1332.5. Furthermore, within a median follow-up duration of 7.3 years, 51 GC deaths were observed. **Table 1** shows the GC-specific mortality in patients with intestinal-type GC according to their general characteristics. The proportion of those who were men (82.0%), aged ≥65 years (41.0%), or those who had smoked in the past (39.9%) was high. Regarding drinking status, the proportion of patients whose daily alcohol intake was <20 g for women and <40 g for men (28.7%) was slightly high. The proportion of cases with middle or high school education (42.1%) and the proportion of cases with underweight or normal weight (44.4%) was also high.

**Table 1:** Observed gastric cancer specific mortality in intestinal-type GC patients with respect to general characteristics.

Characteristics	Intestinal type of GC n (%)	No. of overall death	No. of GC death	Person-years	Median survival years	GC specific survival (%)			
						5-years	10-years	Until final follow up^a^	*p*-value
Overall	178 (100.0)	70	51	1331.5	7.3	77.3	69.0	66.8	–
Sex									
Men	146 (82.0)	59	42	1125.9	8.0	78.8	68.9	66.7	0.776
Women	32 (18.0)	11	9	205.6	6.0	70.0	70.0	70.0	
Ages groups (years)									
<55	47 (26.4)	7	7	417.7	8.9	89.1	83.2	83.2	<0.001
55-64	58 (32.6)	20	14	501.6	8.6	89.5	72.2	72.2	
≥65	73 (41.0)	43	30	412.2	4.8	59.1	57.2	50.9	
Education level									
Elementary school or none	65 (36.5)	30	20	454.7	6.8	70.6	66.6	66.6	0.462
Middle or high school	75 (42.1)	25	18	614.5	8.1	86.3	74.2	69.9	
College or higher	21 (11.8)	8	7	155.6	8.8	70.8	63.8	63.8	
Unknown	17 (9.6)	7	6	106.7	6.2	69.3	62.4	62.4	
Smoking status									
Never	40 (22.5)	15	13	283.9	7.8	71.4	63.9	63.9	0.652
Past	71 (39.9)	30	21	530.8	7.1	75.9	69.0	69.0	
Current	67 (37.6)	25	17	516.8	7.7	82.4	72.6	68.3	
Drinking status^b^ (g/day)									
Never	45 (25.3)	18	17	306.2	6.1	70.8	59.1	59.1	0.013
Past	40 (22.5)	21	14	257.3	7.1	68.4	62.2	51.8	
<20 (g/day) for women or <40 (g/day) for men	51 (28.7)	10	6	483.3	11.4	96.0	86.1	86.1	
≥20 (g/day) for women or ≥40 (g/day) for men	42 (23.6)	21	14	284.7	6.1	70.1	63.9	63.9	
Body mass index (kg/m^2^)									
Underweight or normal weight	79 (44.4)	32	25	581.1	6.9	75.2	66.1	66.1	0.298
Overweight	49 (27.5)	21	17	369.3	7.1	76.9	61.4	55.3	
Obese	39 (21.9)	10	6	322.6	8.8	84.2	84.2	84.2	
Unknown	11 (6.2)	7	3	58.5	2.3	70.0	70.0	70.0	
Registered Hospital									
Chungnam University	80 (44.9)	27	18	681.7	8.9	84.5	75.4	75.4	0.042
Hanyang University Guri	98 (55.1)	43	33	649.8	6.4	71.0	63.6	59.8	
Family history of GC^c^									
No	152 (85.4)	63	47	1114.1	7.0	75.5	66.5	64.1	0.121
Yes	26 (14.6)	7	4	217.4	9.7	87.6	83.0	83.0	
Helicobacter pylori infection									
Negative	66 (37.1)	28	20	509.9	7.8	79.9	68.4	61.6	0.965
Positive	61 (34.3)	21	17	445.3	6.9	72.8	70.8	70.8	
Not performed^d^	51 (28.7)	21	14	376.4	6.8	79.1	67.6	67.6	
Surgery									
No	6 (3.4)	5	5	14.1	0.4	16.7	16.7	16.7	<0.001
Yes	172 (96.6)	65	46	1317.4	7.6	79.4	70.8	68.6	
Adjuvant chemotherapy									
No	138 (77.5)	50	34	1056.1	7.7	79.8	72.8	72.8	0.041
Yes	40 (22.5)	20	17	275.4	5.3	68.6	55.1	48.2	
Stage^e^									
I	113 (63.5)	28	14	996.5	9.7	90.7	85.8	85.8	<0.001
II	25 (14.0)	13	9	169.3	5.9	71.1	59.7	59.7	
III	32 (18.0)	22	21	148.1	4.1	51.8	28.7	14.3	
IV	6 (3.4)	6	6	4.7	0.3	0	0	0	
Unknown	2.0 (1.1)	1	1	13.0	6.5	50.0	50.0	50.0	

^a^The final observation end-point was in 31 December 2016. ^b^This category were divided according to WHO`s recommendation. ^c^Family history of GC included first-degree relatives. ^d^It was impossible to collect the tissue. ^e^Classification by 8^th^ edition of Union for International Cancer Control/American Joint Committee on Cancer (UICC/AJCC) staging system for GC.

The proportion of patients recruited from Hanyang University Guri Hospital (55.1%), those with no family history of GC (85.4%), those with negative *H. pylori* infection (37.1%), those who underwent surgery (96.6%), those who did not receive adjuvant chemotherapy (77.5%), and those with cancer stage I (63.5%) were high.

The survival rate of GC patients according to age group decreased significantly with increasing age (p<0.001). Among current drinkers, the survival rate of GC was significantly higher in those who drank <20 g for women and <40 g for men (p=0.013). In addition, the GC survival rate was high in patients who underwent surgery, did not receive adjuvant chemotherapy, or had low cancer stage. There was no significant difference in GC survival rate according to sex, smoking status, level of education, BMI, family history of GC, and *H. pylori* infection.

### Adjusted hazard ratio for GC mortality according to major prognostic factors

**Table 2** presents the HRs for GC mortality according to the major prognostic factors. The GC mortality rate increased with increasing age in all models. Regarding alcohol consumption, the GC mortality rate decreased when women and men consumed <20 g and <40 g of alcohol, respectively. In addition, the GC mortality rate increased with increasing stage in all models. There were no significant differences in the HRs of GC according to other prognostic factors.

**Table 2:** Adjusted hazard ratios and 95% confidence intervals in the general characteristics.

Characteristics	Model I^1)^	Model II^2)^
	HR (95% CI)	HR (95% CI)
Sex		
Men	1.00	1.00
Women	1.72 (0.80-3.71)	2.55 (0.85-7.68)
Ages (years) (as continuous)
1 years increased	1.06 (1.02-1.10)	1.07 (1.02-1.11)
Education level		
Elementary school or none	1.00	1.00
Middle or high school	0.71 (0.28-1.80)	1.19 (0.41-3.43)
College or higher	0.80 (0.40-1.61)	1.06 (0.47-2.39)
Smoking status		
Never	1.00	1.00
Past	0.69 (0.27-1.75)	0.52 (0.16-1.67)
Current	0.68 (0.26-1.76)	0.70 (0.24-2.04)
Drinking status^a^		
Never	1.00	1.00
Past	1.07 (0.47-2.43)	1.33 (0.52-3.44)
<20 (g/day) for women or <40 (g/day) for men	0.33 (0.12-0.87)	0.31 (0.11-0.90)
≥20 (g/day) for women or ≥40 (g/day) for men	0.93 (0.42-2.06)	1.06 (0.43-2.58)
Body mass index (kg/m^2^)		
Underweight or normal weight	1.00	1.00
Overweight	1.23 (0.63-2.40)	0.98 (0.42-2.29)
Obese	1.35 (0.51-3.58)	1.36 (0.46-4.00)
Registered Hospital		
Chungnam University	1.00	1.00
Hanyang University Guri	0.99 (0.50-1.97)	0.79 (0.32-1.95)
Family history of GC^b^		
No	1.00	1.00
Yes	0.90 (0.30-2.70)	0.94 (0.30-3.02)
Helicobacter pylori infection		
Negative	1.00	1.00
Positive	1.38 (0.69-2.78)	1.44 (0.67-3.09)
Surgery		
No	1.00	1.00
Yes	1.67 (0.21-13.00)	1.79 (0.15-21.71)
Adjuvant chemotherapy		
No	1.00	1.00
Yes	0.97 (0.45-2.09)	0.97 (0.43-2.19)
Stage^c^		
I	1.00	1.00
II	4.03 (1.68-9.70)	4.82 (1.79-12.95)
III	8.13 (3.76-17.58)	9.64 (3.61-25.75)
IV	81.41 (25.93-255.66)	63.81 (7.91-514.41)

^a^This category was divided according to WHO`s recommendation. ^b^Family history of GC included first-degree relatives. ^c^Classification by 8th edition of Union for International Cancer

Control/American Joint Committee on Cancer (UICC/AJCC) staging system for GC.

1)Model I: adjusted for age, sex, adjuvant chemotherapy (no or yes), and stage (I, II, III, IV, and unknown).

2)Model II: model I + further adjusted for body mass index (≤ 22.99, 23.0-24.99, ≥ 25, or missing), education level (≤ middle school, ≥ high school, or missing), smoking status (never, past,

or current smokers), alcohol drinkers (never, past, < 20 g/day for women or < 40 g/day for men, or ≥ 20 g/day for women or ≥ 40 g/day for men), hospital (Chungnam university hospital or

Hanyang university Guri hospital), family history of gastric cancer (no or yes), Helicobacter pylori infection (negative, positive, and not performed), and surgery (no or yes).

### General characteristics by total zinc intakes

**Table 3** shows the general characteristics of patients with intestinal-type GC according to the total zinc intake. Regarding level of education, the proportion of patients with elementary school or no formal education was high (49.2%) in the lowest tertile, whereas the proportion of patients with college or higher was high (49.2%) in the highest tertile (p=0.036). When the energy-adjusted zinc intake increased, the intake of energy-adjusted heme iron (p<0.0001) significantly increased. There was no significant difference according to the tertiles of zinc intake in other variables, such as sex, age, and smoking status.

**Table 3:** Baseline characteristics of patients with the intestinal-type GC according to total zinc intakes.

Characteristics	Total zinc intakes (mg/day)			*p*-value
	T1	T2	T3
Sex, n (%)				
Men	49 (83.1)	49 (81.7)	48 (81.4)	0.968
Women	10 (17.0)	11 (18.3)	11 (18.6)	
Ages groups (years), n (%)				
<55	22 (37.3)	13 (21.7)	12 (20.3)	0.246
55-64	16 (27.1)	21 (35.0)	21 (35.6)	
≥65	21 (35.6)	26 (43.3)	26 (44.1)	
Education level, n (%)				
Elementary school or none	29 (49.2)	25 (41.7)	15 (25.4)	0.036
Middle or high school	5 (8.5)	10 (16.7)	12 (20.3)	
College or higher	17 (28.8)	19 (31.7)	29 (49.2)	
Unknown	8 (13.6)	6 (10.0)	3 (5.1)	
Smoking status n (%)				
Never	10 (17.0)	15 (25.0)	15 (25.4)	0.356
Past	21 (35.6)	27 (45.0)	23 (39.0)	
Current	28 (47.5)	18 (30.0)	21 (35.6)	
Drinking status^a^, n (%)				
Never	11 (18.6)	12 (20.0)	22 (37.3)	0.051
Past	11 (18.6)	12 (20.0)	17 (28.8)	
<20 (g/day) for women or <40 (g/day) for men	21 (35.6)	19 (31.7)	11 (18.6)	
≥20 (g/day) for women or ≥40 (g/day) for men	16 (27.1)	17 (28.3)	9 (15.3)	
Body mass index (kg/m^2^), n (%)				
Underweight or normal weight	26 (44.1)	27 (45.0)	26 (44.1)	0.197
Overweight	16 (27.1)	22 (36.7)	11 (18.6)	
Obese	12 (20.3)	10 (16.7)	17 (28.8)	
Unknown	5 (8.5)	1 (1.7)	5 (8.5)	
Registered Hospital, n (%)				
Chungnam University	27 (45.8)	28 (46.7)	25 (42.4)	0.885
Hanyang University Guri	32 (54.2)	32 (53.3)	34 (57.6)	
Family history of GC^b^, n (%)				
No	51 (86.4)	49 (81.7)	52 (88.1)	0.584
Yes	8 (13.6)	11 (18.3)	7 (11.9)	
*Helicobacter pylori* infection, n (%)				
Negative	18 (30.5)	29 (48.3)	19 (32.2)	0.242
Positive	23 (39.0)	18 (30.0)	20 (33.9)	
Not performed^c^	18 (30.5)	13 (21.7)	20 (33.9)	
Surgery, n (%)				
No	2 (3.4)	1 (1.7)	3 (5.1)	0.587
Yes	57 (96.6)	59 (98.3)	56 (94.9)	
Adjuvant chemotherapy, n (%)				
No	46 (78.0)	45 (75.0)	47 (79.7)	0.827
Yes	13 (22.0)	15 (25.0)	12 (20.3)	
Stage^d^, n (%)				
I	38 (64.4)	33 (55.0)	42 (71.2)	0.380
II	11 (18.6)	10 (16.7)	4 (6.8)	
III	7 (11.9)	15 (25.0)	10 (17.0)	
IV	2 (3.4)	2 (3.3)	2 (3.4)	
Unknown	1 (1.7)	0 (0.0)	1 (1.7)	
Dietary factor (mean ± SD)				
Total energy intake (kcal/day)	1984 ( ± 878.7)	1839 ( ± 730.6)	1877 ( ± 698.3)	0.573
Energy adjusted heme iron intake (mg/day)	0.7 ( ± 0.3)	0.7 ( ± 0.3)	1.1 ( ± 0.5)	<.0001

^a^ This category was divided according to WHO’s recommendation. ^b^Family history of GC included first-degree relatives. ^c^It was impossible to collect the tissue. ^d^Classification by 8^th^ edition of Union for International Cancer Control/American Joint Committee on Cancer (UICC/AJCC) staging system for GC.

### Association between the zinc intake and GC mortality

**Table 4** presents the HRs for GC mortality according to zinc intake in the three adjusted models. Total zinc intake, as a continuous variable and tertile, was significantly associated with lower GC mortality in all models. In the fully adjusted models, the highest tertile of total zinc intake showed a significantly lower GC mortality compared to the lowest tertile (Model III; HR: 0.22, 95% CI: 0.08–0.64). In addition, the tertile of total zinc intake showed a dose-response association with GC mortality (p=0.014).

**Table 4:** Adjusted hazard ratios and 95% confidence intervals for GC specific death.

Zinc intake	No. of GC death	GC Mortality				
		Model I^1)^	Mode1 II^2)^		Mode1 III^3)^	
Total zinc intake						
continuous (mg/day)		0.79 (0.58-1.07)	0.68 (0.49-0.96)		0.66 (0.44-0.98)	
T1	17/59	1.00	1.00		1.00	
T2	22/60	0.92 (0.48-1.77)	0.86 (0.39-1.88)		0.84 (0.38-1.84)	
T3	12/59	0.44 (0.21-0.96)	0.30 (0.13-0.70)		0.22 (0.08-0.64)	
p-trend		0.043	0.007		0.014	
Zinc intake from Staple food						
continuous (mg/day)		0.92 (0.73-1.15)	0.90 (0.71-1.14)		0.74 (0.56-0.99)	
T1	13/59	1.00	1.00		1.00	
T2	26/60	2.41 (1.18-4.94)	2.88 (1.29-6.44)		2.54 (1.11-5.79)	
T3	12/59	0.95 (0.41-2.19)	0.90 (0.37-2.17)		0.71 (0.27-1.86)	
p-trend		0.873	0.981		0.585	
Zinc intake from Animal food groups						
continuous (mg/day)		0.94 (0.76-1.17)	0.88 (0.70-1.11)		0.93 (0.65-1.34)	
T1	17/59	1.00	1.00		1.00	
T2	13/60	0.71 (0.34-1.50)	0.47 (0.20-1.11)		0.51 (0.22-1.22)	
T3	21/59	0.89 (0.45-1.75)	0.70 (0.34-1.46)		0.85 (0.36-1.98)	
p-trend		0.636	0.315		0.607	
Zinc intake from Plant food groups						
continuous (mg/day)		0.83 (0.45-1.56)	0.91 (0.45-1.85)		1.03 (0.50-2.12)	
T1	22/59	1.00	1.00		1.00	
T2	14/60	1.00 (0.48-2.07)	0.91 (0.40-2.07)		0.96 (0.42-2.22)	
T3	15/59	0.73 (0.37-1.45)	0.71 (0.32-1.57)		0.80 (0.35-1.85)	
p-trend		0.626	0.725		0.971	
Zinc intake from Staple food+Animal food groups						
continuous (mg/day)		0.81 (0.61-1.07)	0.63 (0.44-0.90)		0.64 (0.45-0.91)	
T1	17/59	1.00	1.00		1.00	
T2	14/60	0.37 (0.17-0.80)	0.21 (0.09-0.52)		0.19 (0.07-0.47)	
T3	20/59	0.55 (0.27-1.10)	0.31 (0.13-0.73)		0.32 (0.14-0.73)	
p-trend		0.198	0.020		0.027	
Zinc intake from Staple food+Plant food groups						
continuous (mg/day)		0.87 (0.69-1.10)	0.86 (0.67-1.11)		0.74 (0.56-0.98)	
T1	18/59	1.00	1.00		1.00	
T2	22/60	0.94 (0.49-1.79)	1.29 (0.61-2.71)		1.14 (0.53-2.45)	
T3	11/59	0.58 (0.27-1.27)	0.60 (0.26-1.37)		0.42 (0.17-1.04)	
p-trend		0.257	0.319		0.099	
Zinc intake from Staple food+Animal food groups+Plant food groups						1.00
continuous (mg/day)		0.76 (0.58-1.00)	0.62 (0.45-0.86)		0.63 (0.45-0.88)	
T1	19/59	1.00	1.00		1.00	
T2	15/60	0.59 (0.29-1.23)	0.48 (0.21-1.11)		0.48 (0.21-1.11)	
T3	17/59	0.54 (0.27-1.08)	0.38 (0.17-0.85)		0.40 (0.18-0.91)	
p-trend		0.084	0.018		0.028	

1)Model I: adjusted for age, sex, adjuvant chemotherapy (no or yes), and stage (I, II, III, IV, and unknown).

2)Model II: model I + further adjusted for body mass index (≤ 22.99, 23.0-24.99, ≥ 25, or missing), education level (≤ middle school, ≥ high school, or missing), smoking status (never, past, or current smokers), alcohol drinkers (never, past, < 20 g/day for women or < 40 g/day for men, or ≥ 20 g/day for women or ≥ 40 g/day for men), hospital (Chungnam university hospital or Hanyang university Guri hospital), family history of gastric cancer (no or yes), Helicobacter pylori infection (negative, positive, and not performed), and surgery (no or yes).

3)Model III: model II + further adjusted for heme iron or zinc intake.

Zinc intake from staple foods as a continuous variable, was significantly associated with lower GC mortality in model III (HR, 0.74; 95% CI: 0.56–0.99). However, zinc intake from staple foods (tertile) in the second tertile showed a significantly higher GC mortality than the lowest intake in all models. No significant association between zinc intake from animal food groups or plant food groups and GC mortality was observed in any of the models.

In addition, zinc intake from staple and animal food groups, as a continuous variable, was significantly associated with lower GC mortality in Model III (HR, 0.64; 95% CI: 0.45–0.91). In terms of tertile, the highest tertile showed a significantly lower GC mortality compared to the lowest tertile (Model III; HR, 0.32, 95% CI: 0.14–0.73) in model III. The results of zinc intake from staple and plant food groups as a continuous variable were significantly associated with lower GC mortality in model III (HR, 0.74; 95% CI: 0.56–0.98). However, there were no significant results when analyzed according to tertile. The results of zinc intake from the staple, animal, and plant food groups were similar to the results of total zinc intake in all models.

## Discussion

We found an inverse association between zinc intake and GC mortality in patients with intestinal-type GC. Dividing zinc intake according to food sources revealed a significant inverse association between zinc intake from various food sources and GC mortality.

Previous studies reported that dietary factors, such as vitamins, minerals, and natural compounds, may help reduce the risk of chronic diseases, as the main mechanisms of chronic diseases, such as obesity, hypertension, and cardiovascular disease are related to oxidative stress and inflammatory responses (6, 7). In studies related to food intake and chronic diseases, foods containing zinc, such as nuts (8) and the *Nigella sativa* plant (9), were effective against chronic diseases, such as diabetes and cardiovascular disease. However, egg consumption had no effect on hypertension (10). In addition, another review reported that there was insufficient evidence on whether supplementation with vitamins and minerals was effective for cancer and chronic diseases (11).

Several studies have investigated the association between zinc intake and GC. Lee et al. (2005) in the Iowa Women’s Health Study of 41,836 postmenopausal women, reported that the risk of upper digestive tract cancer, including esophageal cancer and GC, decreased as zinc intake increased (RR: 0.13, 95% CI: 0.03–0.63) (15). When they separately analyzed GC and esophageal cancers, the trends were similar for both cancers. In a case-control study conducted in north-west Iran, which is one of the areas with a high incidence of GC, Mohammadreza et al. (2011) reported that the incidence of GC decreased by 53% as zinc intake increased by 5 mg (OR, 0.47; 95% CI: 0.32–0.70) (33). Conversely, in case-control studies in the United States (18) and Italy (19), there was no association between zinc intake and GC incidence.

Few studies have examined the association between zinc intake and mortality. Epstein et al. (2011) reported that zinc intake reduced prostate cancer mortality in a Swedish cohort (34), whereas in a study of Chinese adults, zinc intake increased all-cause mortality, including cancer mortality (35). However, the association between zinc levels and mortality has been inconsistent. This may vary from study to study, depending on zinc intake, source of zinc intake, and cause of death by country. In particular, in a study of Chinese adults with a positive association between zinc intake and mortality (35) reported that in the highest quartile of zinc, more than 40% of the patients had a zinc intake level that was above 150% of the recommended nutrition intake (RNI).

Several mechanisms have been proposed to explain the role of Zn in carcinogenesis. First, zinc has antioxidant and anti-inflammatory properties (36). Zinc delays oxidation in the body and increases the activity of antioxidant enzymes and proteins. It also enhances the expression of zinc finger proteins with anti-inflammatory properties, such as A20 and PPAR-α. Second, zinc regulates immune function (13). A recent review summarized that zinc deficiency reduces the activity of cytokines secreted by T cells and macrophages, NK activity, T-cell differentiation, and the release of certain interleukins and antibodies (37). Third, zinc acts as a tumor suppressor in GC (20). In addition, zinc regulates DNA replication and repair and acts as a structural stabilizing factor in apoptosis (38).

However, these effects may differ depending on the source of the zinc intake. Indeed, in a systematic review and meta-analysis, Li et al. (2014) reported that zinc intake was significantly associated with GC risk in Asia but not in America and Europe (17). The reason for this may be the difference in the source of Zinc in various geographic regions. In Western diets, the main food sources of zinc are animal sources, such as red meat, poultry, and dairy foods, whereas in Asia, the most common source of zinc intake is from grains; moreover, Asians tend to consume more fish and less than red meat (21, 22).

Zinc intake from animal sources, such as meat, have higher bioavailability than those derived from plants as they contain phytic acid, which interferes with zinc absorption (39). However, these animal source foods are also rich in heme iron, which can act as pro-oxidants and may increase the risk of GC (40). Therefore, in some studies (15, 34), heme iron was used as an adjusted variable in models. Thus, we also adjusted for heme iron intake in Model III and found that the association between zinc and GC mortality was more pronounced after adjusting for heme iron.

According to the 2020 Dietary Reference Intakes for Koreans (41), the recommended intake of zinc for men and women aged 19–64 years is 10 mg and 8 mg, respectively, and for men and women aged ≥65 years is 9 mg and 7 mg, respectively. In our cohort study, we found that zinc intake was 8.83 ± 3.64 mg/day and energy-adjusted zinc intake was 9.30 ± 1.06 mg/day. Zinc intake in men and women was similar. The patients in our cohort appeared to consume zinc at levels similar to the recommended intake for Koreans. A review study suggested that adequate intake of zinc has beneficial effects on health as zinc is involved in antioxidant and immune responses, as described above; however, excessive amounts of zinc may have a negative effect on health due to its immunosuppressive effect (37). Therefore, research on adequate zinc intake in each population group and in various diseases, such as cancer is necessary.

Furthermore, the zinc contents of one serving size in Korean diets were as follows: 12.72 mg/80 g of oyster, 2.56 mg/60 g of beef, 1.85 mg/90 g of whole grains, 1.44 mg/80 g of shrimp, 1.28 mg/60 g of pork, and 1.12 mg/80 g of squid (41). However, since oysters and beef are not frequently consumed by Koreans daily, the staple foods contributing to the highest zinc intake of Koreans are daily food groups, such as rice and noodles. We presented the results of zinc from staple food groups, animal food groups, and plant food groups, separately or in combination, which have not been presented in other studies. Although no meaningful results were obtained when we analyzed each food group, when zinc intake from staple, animal, and plant food sources were combined, the significance between zinc and GC mortality was most pronounced. When the source of zinc intake is obtained through various foods, it may be effective in reducing mortality by balancing it with other nutrients. The intake of zinc from animal sources, such as red meat, may be effective in terms of bioavailability. However, animal-based foods also contain nutrients that are negative for GC. Indeed, Koreans consume zinc through regular daily diets, such as through whole grains or grains. In addition, a balanced intake of zinc from animal sources, such as squid and fish, as well as from plant sources, such as fruits and vegetables, may be beneficial to GC mortality.

Lim et al. (2012) reported that male Korean patients had more dietary habit problems and unbalanced nutrition intake than female Korean patients (42). Thus, they suggested the importance of education and management of proper nutrition, particularly in men. Since intestinal-type GC is particularly common in older men and is greatly affected by environmental factors, such as diet, continuous nutrition education is needed to reduce the incidence of GC and mortality in these vulnerable groups. In addition, since the rate of malnutrition is high among patients with upper gastrointestinal cancer (43), proper nutritional status and supplementation are very important to improve their prognosis.

Our study has several strengths. First, the temporal relationship between dietary factors and mortality is clear because our study prospectively followed mortality after collecting lifestyle and dietary factors before GC diagnosis. Second, we only included patients with intestinal-type GC, which is highly diet-related. To the best of our knowledge, this is the first study to confirm the association between zinc intake and mortality in patients with intestinal-type GC. Third, we adjusted for the latest cancer stage, which is the strongest prognostic factor for death and used the Union for International Cancer Control (UICC)/AJCC 8^th^ edition staging system. However, this study had some limitations. First, the sample size was small because we only selected patients with intestinal-type GC. Second, because dietary information was obtained by recall, the possibility of recall bias could not be excluded. However, in spite of this limitation, a study previously reported that the FFQ results, which recalled the patients’ diets 10 years prior, were also reliable (44). In addition, a study on the validity of the FFQ reported that the reproducibility and validity of the FFQ performed at 3-year intervals were acceptable (45). Third, it is possible that the dietary habits and lifestyles of patients changed after the diagnosis of GC. However, an additional follow-up of dietary changes was not performed. Meanwhile, nutritional status is more likely to deteriorate as the GC stage worsens (46). Thus, we adjusted the GC stage to reflect the nutritional status after GC surgery.

In conclusion, our results suggest that zinc contributes to improved survival of patients with intestinal-type GC in Korea. Zinc intake from various food sources may help increase the survival rate of patients with GC, and continuous nutrition education and management are required in those who are prone to nutritional imbalance. Further research is warranted to confirm the association between zinc intake and GC mortality in larger samples.

## Data availability statement

The datasets presented in this article are not readily available because of patient privacy. Requests to access the datasets should be directed to the corresponding authors.

## Ethics statement

The studies involving human participants were reviewed and approved by the institutional review board (IRB) of Hanyang University Guri Hospital (IRB no. 2003-4). For follow-up, additional approval was obtained from the IRB of Chungnam National University Hospital (IRB no. CNUH 2017-12-039), and the IRB of Hanyang University Guri Hospital (IRB no. 2018-01-021-001). The patients/participants provided their written informed consent to participate in this study.

## Author contributions

Data analysis: JHK. Writing - original draft: JHK. Writing - review and editing: HJK. Data collection, patient recruitment, and data management: CHP, CSE, DSH, YSK, KSS, BYC, and HJK. Funding acquisition: HJK. Supervision: HJK and BYC. All authors contributed to the article and approved the submitted version.

## Funding

This research was supported by the Basic Science Research Program through the National Research Foundation of Korea (NRF) funded by the Ministry of Education (Grant Number 2020R1I1A3A04036989).

## Acknowledgments

We are very grateful to all patients who participated in this study and the hospital staff who contributed to the study procedure.

## Conflict of interest

Author YSK was employed by PDXen Biosystems Co.

The remaining authors declare that the research was conducted in the absence of any commercial or financial relationships that could be construed as a potential conflict of interest.

## Publisher’s note

All claims expressed in this article are solely those of the authors and do not necessarily represent those of their affiliated organizations, or those of the publisher, the editors and the reviewers. Any product that may be evaluated in this article, or claim that may be made by its manufacturer, is not guaranteed or endorsed by the publisher.
